# Surgery for patellar dislocation has evolved towards anatomical reconstructions with assessment and treatment of anatomical risk factors

**DOI:** 10.1007/s00167-020-06277-x

**Published:** 2020-09-18

**Authors:** Mikko M. Uimonen, Jussi P. Repo, Tuomas T. Huttunen, Heikki Nurmi, Ville M. Mattila, Juha Paloneva

**Affiliations:** 1 Department of Surgery, Central Finland Hospital, Keskussairaalantie 19, 40620 Jyväskylä, Finland; 2 Department of Emergency, Anaesthesia and Pain Medicine, Tampere University Hospital, Tampere, Finland; 3 Faculty of Medicine and Health Technology, Tampere University, Tampere, Finland; 4 Department of Orthopaedics and Traumatology, Tampere University Hospital, Tampere, Finland; 5 COXA Hospital for Joint Replacement, Tampere, Finland; 6 University of Eastern Finland, Kuopio, Finland

**Keywords:** Patellar dislocation, Patellar dislocation surgery, Incidence, Knee injury

## Abstract

**Purpose:**

Increasing knowledge on the treatment of patellar dislocation has resulted in the development of new surgical techniques for patella stabilisation. National incidence and trends in surgery for patellar dislocation were examined using data from the Finnish National Hospital Discharge Register (NHDR). The hypothesis was that an increased understanding of the pathophysiology of patellar instability has increased the popularity of reconstructing damaged structures and modification of anatomical risk factors.

**Methods:**

Data from the years 1997–2016 were collected from the NHDR database using ICD-10 diagnostic codes and the Nomesco Classification of Surgical Procedures (NCSP) codes. Surgical procedures were categorised into subgroups representing the main surgical approaches of patellar dislocation. Total incidence of surgery for patellar dislocation and change in incidence during the study period were calculated.

**Results:**

A total of 9702 operations for patellar dislocation were performed during the study period. Median (IQR) patient age at time of primary surgery was 23 (18–34) years. The total incidence of surgeries remained stable across the study period at of 8.9 per 100,000 person-years. Incidences of ligament reconstruction, femoral osteotomies and osteochondral fragment reimplantation operations multiplied during the study period. Ligament reconstruction procedures were the most performed operations at the end of the study period.

**Conclusion:**

The incidence of surgical procedures for patellar dislocation remained unchanged during the years 1997–2016. Ligament reconstruction procedures increased in popularity. Surgical techniques have shifted towards the reconstruction of damaged structures and the modification of congenital anatomical risk factors for patellar dislocation. Diversified surgical techniques have enabled the tailoring and combining of stabilizing procedures according to the patient’s individual anatomy.

## Introduction

Lateral patellar dislocation is a common knee injury in the young, active population [3, 17, 35]. Indeed, previous studies have found the incidence of acute patellar dislocation to be between 23 and 77 per 100,000 person-years, depending on the demographics of the target population [21, 42, 45]. Peak incidence, estimated to be 148 per 100,000 person-years, occurs between the ages of 14 and 18 [43]. The incidence has been shown to decline with age [43]. Among the female population, this decline begins at an earlier age (14–18 years) than among the male population (19–25 years) [43]. According to the findings of a 10-year follow-up study, 23% of patients with patellar dislocation suffered a recurrent dislocation [21]. The risk of recurrent dislocation is even higher in skeletally immature patients, among whom the incidence of recurrence during a 10-year follow-up study was reported to be 45% [42].

Based on clinical experience, primary acute patellar dislocation is generally treated conservatively. However, in cases of osteochondral fracture, where a loose fragment of bone is detected inside the knee joint, surgery is the first-line treatment [47, 48]. If patellar dislocation recurs, surgical patellar stabilisation may be indicated [47]. At present, however, consensus on the best treatment recommendations and guidelines for primary patellar dislocation is lacking [47]. A recent meta-analysis by Yang et al. comprising 16 randomised controlled trials (RCT) or cohort studies published between 1986 and 2018 cautiously concluded that surgical treatment might also be more favourable than conservative treatment for patients with their first patellar dislocation [53]. This conclusion was based on the finding that surgically treated patients had higher Kujala scores (anterior knee-specific patient-reported outcomes) and a lower rate of re-dislocations compared with conservatively treated patients. Nevertheless, due to the rapid development of surgical techniques during the past few decades, the conclusion was based on heterogenous study populations. Previous results have indicated that not all surgical techniques are as equally effective [2, 5, 46, 50, 53].

The purpose of the present study was to investigate trends in the surgical treatment of patellar dislocations between the years 1997 and 2016 using nationwide data from the Finnish population-based register of medical treatment. The hypothesis was that an increased understanding of the pathophysiology of patellar instability has led to an increase in the popularity of the reconstruction of damaged structures and the modification of anatomical risk factors. Moreover, knowledge on current trends in the surgical treatment of patellar dislocation in relation to recent research may provide an important insight into the future prospects of different approaches to the surgical management of patellar dislocation.

## Materials and methods

Based on the Medical Research Act (488/1999), ethical approval is not required for register-based studies in Finland. Data were extracted from the Finnish National Hospital Discharge Register (NHDR, National Institute of Health and Welfare, Finland). Information on hospital admissions (both inpatient and outpatient) and treatment events in every Finnish hospital are recorded in the NHDR. The NHDR contains data on patient age and sex, length of hospital stay, patient’s domicile, and diagnoses and procedures performed during the hospital stay. The register is mandatory for all private and public sector hospitals in Finland. The coverage and accuracy of the NHDR for surgical treatment have proven to be excellent [24, 31, 49].

All patients who had undergone surgery for patellar dislocation between 1 January 1997 and 31 December 2016 were included in the study. A data search was conducted using the codes S83.0, M22.0 and M22.3 from the International Classification of Diseases, 10th revision (ICD-10, WHO) and applicable codes from the Nomesco Classification of Surgical Procedures (NCSP) (Finnish version). Combining the procedure code with patellar dislocation diagnosis in patient selection was assumed to mitigate the bias caused by the fact that the same procedure codes may apply in diverse procedures. Information was obtained on age, gender, diagnoses and the surgical procedures performed.

To better focus on the most relevant procedures, those procedures performed fewer than 100 times during the study period were excluded from the analysis. In total, 16 surgical procedure codes for patellar dislocation were selected for review. The codes were then grouped as presented in Table 1. Because the procedure codes used in patellar dislocation surgery are not completely specific, explanations of the procedures included for each group were made based on clinical experience.

**Table 1:** Classification of procedure codes and explanations of the procedures in each class

Procedure codes	Includes
Ligament reconstruction or repair	
NGE60; NGE65	MPFL or MPTL reconstruction or repair
Extensor realignment	
NGL66; NGL30; NGK99	Tibial tubercle transfer Patellar tendon shift Imbrication of vastus medialis
Trochleoplasty	
NGG00	Trochleoplasty
Femoral osteotomies	
NFK30	Varus-producing osteotomy of femur Torsional osteotomy of femur
Reimplantation of osteochondral fractures	
NGF30; NGF35	Open or arthroscopic reimplantation of osteochondral fractures
Debridement	
NGA30; NGD05; NGF00; NGF25	Arthroscopy Loose fragment removal Plica resection Menisci debridement
Capsular release	
NGE10; NGE15	Lateral capsular release
Unspecified procedures	
NGH20	

The included procedure codes were used together with patellar dislocation diagnosis. Each included procedure code was used more than 100 times during the study period

### Statistical analysis

The total and annual incidences of surgical procedures for patellar dislocation were calculated using annual population register data published by Statistics Finland (https://www.stat.fi/index_en.html). The total incidence of operations was calculated by extracting from the NHDR all the surgical operations for patellar dislocation identified by applying the relevant diagnostic and procedure codes. In calculating the total incidence of operations, an individual operation was counted as one, even if more than one procedure code was recorded in the same operation. In addition, the incidence of each procedure type was examined separately. In contrast to the total incidence calculation, when calculating the incidence of each procedure type, each procedure code was counted as one also in cases where a single operation was recorded under more than one procedure code. Total incidence was reported per 100,000 person-years and annual incidence per 100,000 persons. As the register data used in incidence calculations were based on the total population of the country, the resulting total and annual incidences of each procedure represent the genuine incidence. Thus, no statistical estimation methods, such as 95% confidence interval calculations, were used. The analysis was performed using R 3.6.1 statistics software [40].

## Results

**Fig. 1:**
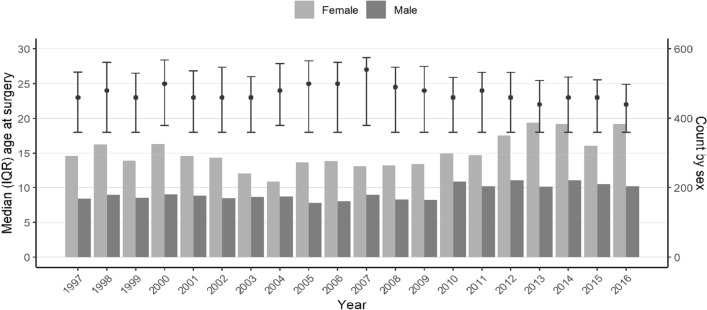
Age and sex distributions of the patients during the study period. Points represent median age at the time of the surgery and whiskers show the interquartile range. Bars represent annual count of males and females undergoing surgery

**Fig. 2:**
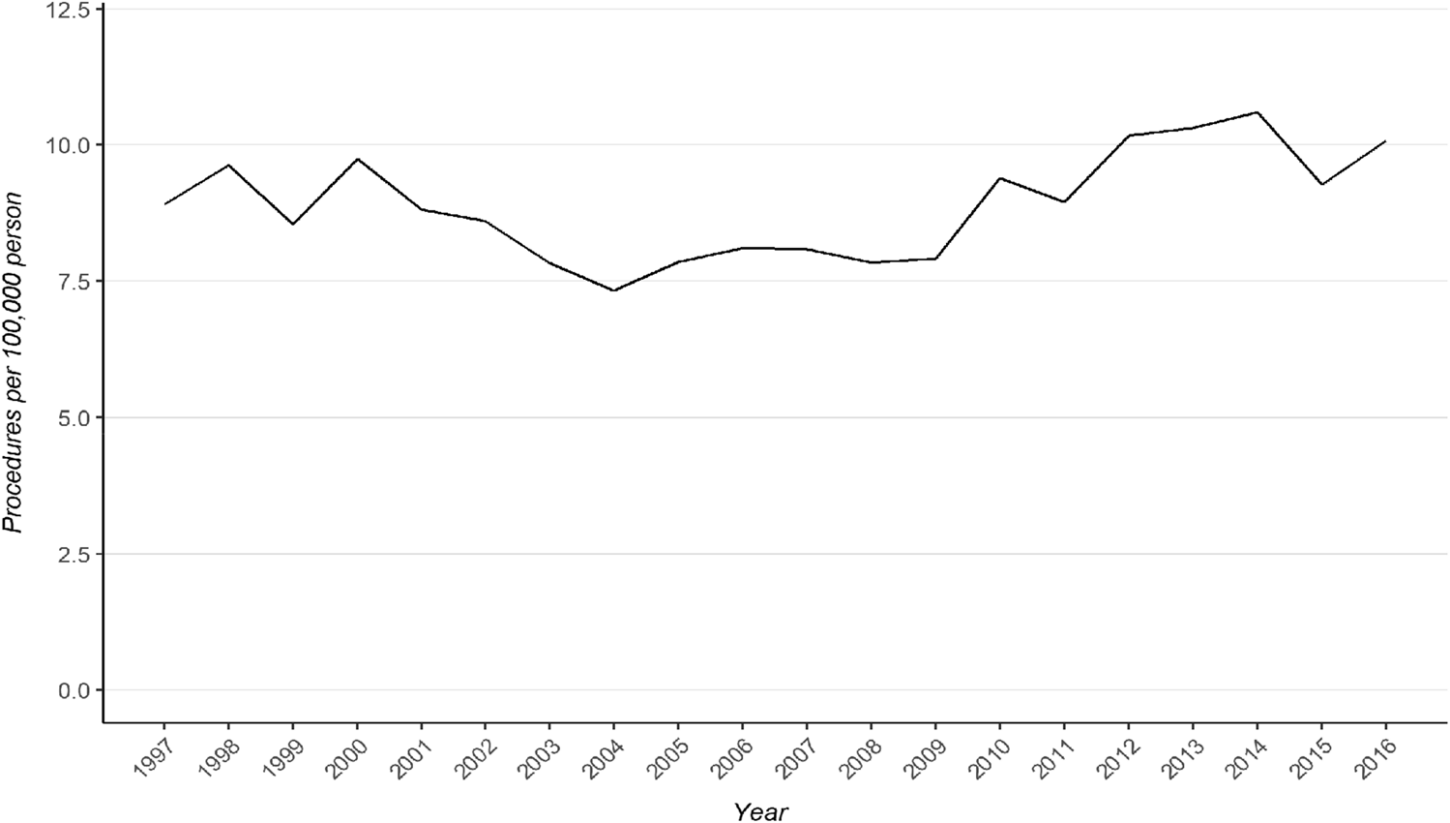
Incidence of surgical operations for patellar dislocation from 1997 to 2016

By applying the relevant procedure codes, 9702 surgical operations for patellar dislocation were identified in 8 333 patients during the study period. Of these, 61% were female (*n* = 5 071). Median (IQR) patient age at the time of surgery was 23 (18–34) years, ranging from 4 to 88 years. The ages and sex distribution remained stable across the study period (Fig. 1). The incidence of surgical procedures for patellar dislocation remained stable between the years 1997 and 2016 (8.9 in 1997 vs. 10.1 in 2016; see Fig. 2) with a total incidence of 8.9 per 100,000 person-years.

**Fig. 3:**
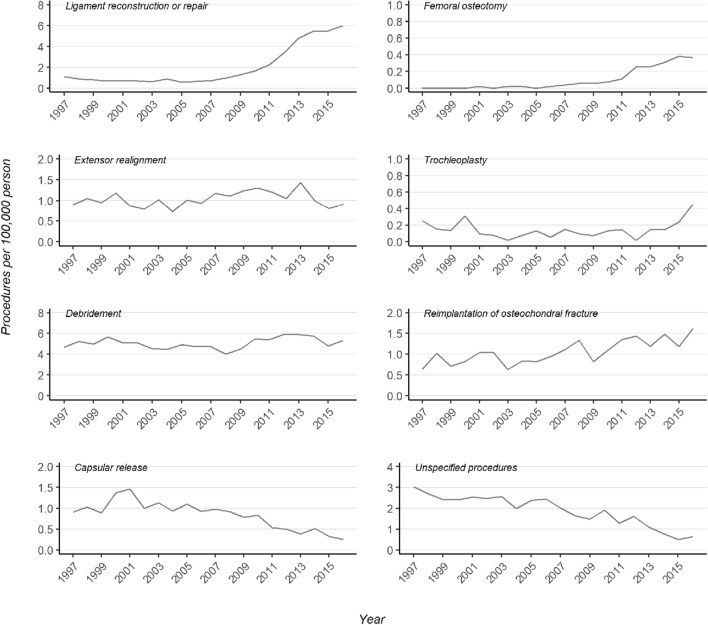
Trends in different procedure types

At the end of the study period, the most prevalent patellar dislocation operations were ligament reconstructions (Table 2). The incidence of ligament reconstruction procedures accelerated rapidly after the year 2009 and continued to rise until the end of the study period (Fig. 3). In turn, the incidence of debridement procedures, which had been the most performed procedures, remained stable across the study period. The incidence of ligament procedures overtook debridement procedures after the year 2015. The most prominent change occurred in the incidence of femoral osteotomy procedures. The rise in popularity of these procedures began after 2011. Thereafter, the incidence rose by 227% until the end of the study period (Fig. 3). The incidence of trochleoplasties forms a slightly U-shape curve with higher incidence at the beginning of the study period followed by a lower incidence until 2014. Subsequently, the incidence rose towards the end of 2016. The incidence of femoral osteotomy and trochleoplasty procedures was, however, still low when compared to the other procedures. Furthermore, the incidence of procedures concerning the reimplantation of osteochondral fragments after patellar dislocation rose steadily across the study period with a total rise of 152%. Extensor realignment procedures remained stable with only a 2% increase. On the other hand, the incidence of capsular release procedures decreased after 2001 with a total decrease of 82% until the end of the study period. In addition, the popularity of the procedure code for unspecified knee dislocation procedure (NGH20) decreased 79% across the study period.

**Table 2:** Incidence and percentage change by procedure type

	Incidence per 100,000 persons		Total change, %
	1996	2016	
Ligament reconstruction or repair	1.11	6.00	+ 442
Femoral osteotomy	0.00	0.36	NA
Extensor realignment	0.89	0.91	+ 2
Trochleoplasty	0.25	0.45	+ 80
Debridement	4.68	5.31	+ 13
Reimplantation of OCF	0.64	1.62	+ 152
Capsular release	0.91	0.25	− 72
Unspecified procedures	3.03	0.64	− 79

*OCF* osteochondral fracture, *NA* non-applicable

## Discussion

The main finding of this study on patellar dislocation surgery, using nationwide register data, was that the total incidence of surgery for patellar dislocation has remained relatively stable. However, the incidences of ligament reconstruction and repair procedures, femoral osteotomies and the reimplantation of osteochondral fragments increased during the study period. However, the incidence of debridement and extensor realignment procedures have remained unchanged and the incidence of lateral releases has decreased. The peak incidence of patellar dislocation has been reported to occur between the ages of 14 and 18 [43]. In the current study, the median age of the patients was 23 with no prominent changes across the study period.

### Evolution of patellar dislocation surgery

During the twentieth century, the most common surgical procedures for the treatment of patellar instability were probably those originally described by Roux and Goldthwait, Galeazzi et al. and Hauser et al. In the late nineteenth century, Roux and Goldthwait, presented a technique in which the lateral part of the patellar tendon is transferred medially to enhance the medial support of the patella [19, 22, 41]. Later, in the early twentieth century, Galeazzi et al. introduced a medial patello-tibial ligament reconstruction technique using a semimembranosus tendon graft [18]. Hauser et al. increased the repertoire of techniques for managing patellar instability with a technique combining tibial tubercle distalisation and medialisation with the release of the lateral retinaculum and imbrication of the vastus medialis tendon [22]. In the late twentieth century, arthroscopic procedures enabling mini-invasive surgical procedures, such as isolated arthroscopic lateral capsular release and arthroscopic debridement procedures, were introduced [13]. Open stabilising techniques, such as torsional osteotomies of the femur or tibia, trochleoplasty, tibial tubercle transfer and medial patello-femoral ligament (MPFL) reconstruction, have also been developed during the past three decades [4, 8, 15, 16, 29, 30, 39, 44]. Today, thanks to a greater knowledge of natural history and the anatomical factors associated with patellar instability as well as increasing expertise in management techniques for individual anatomic abnormalities and technological advancements, treatment can be increasingly tailored to the individual patient’s anatomy [10].

### Recent patellar dislocation surgery trends

According to the results of the present study, ligament reconstruction or repair procedures have become the gold standard in patellar dislocation surgery. The increase in these procedures took place during the years 2007–2009 and was preceded by a substantial increase in research on MPFL reconstruction [6, 7, 12, 36, 37]. Since Gomez et al. introduced the MPFL reconstruction technique using a synthetic polyester ligament graft [20] and Avikainen et al. presented their technique using abductor magnus tenodesis [4], the number of studies on MPFL reconstruction in cases of patellar instability have increased. The results of these studies have consistently shown efficacy in the reduction of recurrent patellar dislocations in patients receiving MPFL reconstruction compared to those treated non-surgically [2, 47, 53, 54]. However, functionality outcomes have shown more inconsistent results [2, 47, 53, 54]. In the literature, MPFL reconstruction continues to be the most recommended surgical management technique for patellar dislocation. Moreover, if the patient has anatomical risk factors for patellar dislocation, the risk for recurrent dislocation remains high even after successful MPFL reconstruction [26]. Thus, to manage the anatomical risk factors, MPFL reconstruction alone might not be enough and should instead be combined with bony procedures [26, 33, 34].

During last 2 decades, femoral osteotomy procedures for patellar instability caused by axial deformities of the lower extremity have been developed. These techniques include varus-producing osteotomy for correcting excessive valgus malalignment of the knee joint [29, 39] and torsional osteotomies of the femur for correcting torsional malalignment [8, 16]. In the current study, the rise in incidence of these osteotomies were seen after 2011. The present findings suggest that femoral osteotomies are becoming more widespread in patients with patellar instability and may provide an additional approach to ligament reconstruction procedures.

Remodelling of the femur trochlea was originally introduced in the early twentieth century by Albee et al. who presented lateral facet elevation trochleoplasty [1]. During the 1970s and 80 s, Masse et al. presented a new trochleoplasty technique, with deepening of the trochlear sulcus, that was later modified by Dejour et al. [15, 30]. Currently, the main indication for trochleoplasty is dysplastic trochlea, which predisposes the patella to dislocate laterally. Research on the outcomes of trochleoplasty procedures increased during the early 2000s with promising results [14, 38, 50, 52]. According to the results of the current study, the popularity of trochleoplasty remained relatively low across the study period until 2014, from which point the popularity appeared to slightly increase towards the end of the study.

While procedures focusing on the ligaments and bony structures of the patellofemoral joint have considerably increased in number in the current study, a decrease was seen in extensor realignment procedures. It is likely therefore that more recent methods for improving extensor apparatus tracking, such as torsional osteotomy, have offered an option for extensor realignment procedures.

In contrast to debridement procedures, the reimplantation of osteochondral fractures increased during the study period. Studies on the long-term outcomes of patellar dislocation have shown a marked increase in incidence of osteoarthritis in cases where an articular cartilage defect is present in the knee joint [23, 28, 32]. These findings might have led surgeons to favour reimplantation rather than the removal of loose fragments. On the other hand, the development of arthroscopic technology and reimplantation techniques during the study period has also facilitated reimplantation efforts [11]. New reimplantation techniques and tools, such as biodegradable pins and sutures, have been developed and applied in practice [25, 27, 51, 55]. In addition, the increase in reimplantation may reflect technological advancements on a larger scale in knee surgery than in only patellar dislocation surgery [9].

The findings of the present study suggest that surgery for patellar dislocations has evolved towards the reconstruction of damaged structures and the active modification of congenital anatomical risk factors for patellar dislocation. Moreover, techniques have progressed in the wake of expanding research interest. Knowledge has been gained on anatomical risk factors and several methods for correcting these malformations have been developed. This rapid development was reflected in the change in the trends of surgery for patellar dislocation in the 2010s.

It should be noted that even though surgery trends have followed the research literature, the outcomes of the change remain unclear. Changes in the incidence of surgical procedure do not directly reflect the efficacy of the given procedure. Indeed, there might be other factors behind the change, such as the implementation of more rigorous and precise criteria for performing the procedure. In future studies, it would be essential to examine the influence of combining and tailoring methods according to the patient’s individual anatomy with the outcomes of patellar dislocation surgery, such as recurrence of patellar dislocations as well as in reoperations due to patellar instability. Prospective, ideally randomised, controlled studies comparing the latest surgical techniques are therefore advocated.

### Strengths and weaknesses

Clearly, a strength of the present study is the availability of an accurate, nationwide population-based sample covering all surgical operations for patellar dislocation in Finland during the 20-year study period. The authors are unaware of a previous study that has published on the national incidence of surgery for patellar dislocation. Previous studies have shown the NHDR to be accurate and to have good coverage of surgical treatment in Finland. Therefore, the present results should be interpreted as a good description of clinical reality [24, 31, 49]. The main limitation of the current study is the inability of the NSPC procedure coding system to adequately differentiate procedures. Many procedure codes, for example, those used in MPFL reconstruction procedures, are not completely specific and accurate. Therefore, the codes used may include completely different techniques, such as reconstruction and repair, and also include procedures on other ligaments. In order to mitigate the given bias, procedures that were performed fewer than 100 times during the study period were excluded. In addition, only those patients who had undergone surgery were included in the current study. Thus, patellar dislocation and instability patients that did not undergo surgery were automatically excluded. As the decision on the surgical treatment of patellar instability is made by the treating surgeon based on clinical and radiological examination, it is unlikely that patients with no effective episodes of dislocation end up in surgical treatment. In some cases, however, it is possible that if MRI clearly reveals anatomical risk factors for patellar instability and dislocation. Thus, the surgeon might decide to operate the knee due to recurrent patellar instability symptoms, even though no effective episodes of dislocations have occurred. It is therefore possible that patients with no effective episodes of patellar dislocations have also been included in the current study. Finally, the NHDR does not differentiate the side of operated knee, which impeded the examination of the reoperation rate and the techniques used in subsequent operations. Therefore, the approach of the current study was to observe general trends in single procedures performed for patellar dislocation rather than the treatment of individual patients.

## Conclusion

Although the incidence of surgical operations for patellar dislocation has remained stable over the past two decades, a change in surgical management techniques has occurred. There has been a shift towards the reconstruction of damaged structures and the modification of congenital anatomical risk factors for patellar dislocation. In clinical practice, therefore, the findings of the current study may encourage clinicians to tailor and combine surgical techniques according to the patient’s individual anatomy and injury characteristics.
